# LncRNA ANRIL/miR-7-5p/TCF4 axis contributes to the progression of T cell acute lymphoblastic leukemia

**DOI:** 10.1186/s12935-020-01376-8

**Published:** 2020-07-23

**Authors:** Gang Li, Lan Gao, Jing Zhao, Dejun Liu, Hui Li, Min Hu

**Affiliations:** Department of Clinical Laboratory, Henan Provincial People’s Hospital, People’s Hospital of Zhengzhou University, Weiwu Road, No. 7, Zhengzhou, Henan 450003 China

**Keywords:** T-ALL, lncRNAs, ANRIL, miR-7-5p

## Abstract

**Background:**

Antisense non-coding RNA in the INK4 locus (ANRIL) is of great importance in cell biological behaviors, and ANRIL functions in many kinds of cancers including leukemia. However, the mechanism of ANRIL in the progression of T-cell acute lymphoblastic leukemia (T-ALL) has not been clarified clearly.

**Methods:**

qRT-PCR was performed to detect ANRIL expression in T-ALL samples. T-ALL cell lines (MOLT4, CCRF-CEM and KOPT-K1) were used as the cell models. The function of ANRIL on T-ALL cells was investigated by CCK-8 assays, Transwell assays, and apoptosis experiments in vitro. qRT-PCR, Western blot, luciferase reporter assay and RIP assay were used to confirm the interactions between ANRIL and miR-7-5p, miR-7-5p and its target gene transcription factor 4 (TCF4).

**Results:**

ANRIL was significantly up-regulated in T-ALL samples. Its knockdown markedly inhibited viability, migration and invasion of T-ALL cells, but its overexpression exerted the opposite effects. TCF4 was proved to be a target gene of miR-7-5p. ANRIL down-regulated miR-7-5p via sponging it and in turn up-regulated TCF4.

**Conclusions:**

LncRNA ANRIL can modulate malignant phenotypes of T-ALL cells, possibly by regulating miR-7-5p/TCF4 axis, and it serves as a potential therapeutic target for T-ALL.

## Background

Acute lymphocytic leukemia (ALL), a common hematological malignancy among children, accounts for 80% of the leukemia cases in children, and it also ranks the second most common acute leukemia in adults [1–3]. The 5-year survival rate in adults is approximately 30%–50%, and that in children is about 90% [4, 5]. Patients with T-ALL suffer from high risks of recurrence due to acquired drug resistance [6]. The current treatment options for these patients are limited [7]. Hence, it is of great importance to probe the mechanism of T-ALL progression and provide new clues for the development of new drugs.

Long non-coding RNAs (lncRNAs) is deemed as a sort of endogenous non-protein coding transcripts exceeding 200 nucleotides in length [8]. More and more evidence reveals that lncRNAs participate in the growth and development of the human body as well as the tumorigenesis and progression of tumors [9, 10]. LncRNAs can work as oncogenes or tumor suppressors in various tumors, and their abnormal expression levels can be used as indicators for tumor occurrence, metastasis or recurrence [11, 12]. An enormous number of studies authenticate that the dysregulation of antisense non-coding RNA in the INK4 locus (ANRIL) is related to the progression of multiple cancers [13–17]. For example, ANRIL promotes tumorigenesis through up-regulation of EGFR1 expression in head and neck squamous cell carcinoma [16]; knockdown of ANRIL restrains the proliferation, migration and invasion of liver cancer cells [17]. Besides, the significance of ANRIL in leukemia becomes increasingly prominent [18, 19]. For instance, it is reportedly confirmed that ANRIL promotes proliferation and inhibits apoptosis of adult T-cell leukemia cells through cooperating with EZH 2 to activate NF-κB pathway [19]. However, ANRIL’s role in T-ALL and its mechanisms need further investigation.

MicroRNAs (miRNAs), small non-coding RNAs containing 18-24 nucleotides, are endowed with the ability of post-transcriptional regulation via binding specifically to the 3′-untranslated region (3′-UTR) of target genes [20, 21]. Accumulating studies indicate that miRNAs participate in a diversity of cellular biological activities [22–24]. More than 50% of miRNA, situated in cancer-related genomic regions, functions in carcinogenesis or tumor suppression [25]. MiR-7 can suppress BCR-ABL and inhibit the activity of PI3K/AKT pathway, suggesting that miR-7 exerts the anti-cancer effect in chronic myeloid leukemia [26]. MiR-7-5p is a tumor suppressor in diverse cancers [27–31]. In gastric cancer, miR-7-5p suppresses the metastasis of cancer cells via inhibiting epidermal growth factor receptor signaling pathway [30]. In melanoma, miR-7-5p can repress the growth and invasion of cancer cells via inhibiting the RelA/NF-κB signaling pathway [31]. But little is known concerning whether miR-7-5p has an inhibitory effect on T-ALL and the regulatory mechanism.

Transcription factor 4 (TCF4) is abnormally expressed in many tumors, such as pancreatic cancer and leukemia [32–34]. TCF4 is fundamental to the biological behavior of cancer cells, targeting multiple oncogenes, like MYC, PRMT 5, CCND 1, CD44 and MMP-2, all of which are involved in the progression of tumors [35–40]. However, the role of TCF4 and its upstream regulatory mechanism in the progression of T-ALL are obscure.

Bioinformatics suggests that there are potential binding sites between ANRIL and miR-7-5p, miR-7-5p and 3′UTR of TCF4. This work aimed to probe into the function of ANRIL/miR-7-5p/TCF4 axis in T-ALL and provide a theoretical foundation for the progression of T-ALL.

## Materials and methods

### T-ALL patients and ethics statement

Clinical specimens (bone marrow tissues) were obtained from 27 patients newly diagnosed as T-ALL. Bone marrow tissues from 27 healthy cases served as the control group. All participants in this study signed informed consent, and the study obtained the approval of the Clinical Ethics Committee of Henan Provincial People’s Hospital. The inclusion criteria for each patient were as follows: newly diagnosed T-ALL patients who visited Henan Provincial People’s Hospital from January 2015 to March 2018 were included in the study; these patients were diagnosed according to classification of morphology, immunology, cytogenetics, and molecular biology. The exclusion criteria were: severe heart dysfunction, severe arrhythmia, severe lung dysfunction, severe hepatic or renal dysfunction; other solid tumor history. For T-ALL patients, flow cytometry analysis was used to make diagnosis and differentiation (positive for T cell antigens like CD2, CD3, CD4, CD7, CD8, positive for stem cell/progenitor cell marker CD34 and CD38, and negative for myeloid antigens like CD13, CD14, CD15, CD33 and CD117). The patients’ group: 21 males and 6 females, with a median age of 28 years (range: 11–47 years). The healthy controls’ group: 17 males and 10 females, with a median age of 26 years (range: 7–49 years). The samples were obtained at the time of the diagnosis, and the enrolled patients had not received cancer-related treatments.

### Cell culture

Human T-ALL cell lines (MOLT4 cells, CCRF-CEM cells, and KOPT-K1 cells) were available from the Cell Bank of the Chinese Academy of Sciences (Shanghai, China). T lymphocytes from normal bone marrow tissues were used as normal control. All T-ALL cells were cultured in RPMI-1640 medium (Invitrogen, CA), supplemented with 10% fetal bovine serum (FBS, Hyclone, South Logan, UT) and 1% penicillin/streptomycin (Sigma-Aldrich, St. Louis, MO, USA) in 5% CO_2_ at 37 °C.

### RNA isolation and quantitative real-time polymerase chain reaction (qRT-PCR)

Total RNA was isolated using TRIzol agent (Invitrogen, Carlsbad, CA, USA) according to the manufacturer’s instruction. RNA was synthesized into complementary DNA using a Prime Script RT kit (Takara, Otsu, Japan). ANRIL, miR-7-5p and TCF4 expression was detected by ABI 7500 Real-Time PCR system (Applied Biosystems, USA). qRT-PCR was performed with the following cycling conditions: denaturation at 95 °C for 30 s, annealing at 60 °C for 30 s and extension at 72 °C for 15 s, 40 cycles. GAPDH or U6 were used as internal references. Specific PCR primers were synthesized by Thermo Fisher Scientific (Shanghai, China). Primer sequences in this study were shown in Table 1. The relative expressions of the genes were calculated utilizing the 2^−ΔΔCt^ method.

**Table 1:** The sequence for PCR primer sequences and ANRIL shRNA and control shRNA

Gene	Sequence
*ANRIL*	F, 5′-CAACATCCACCACTGGATCTTAACA-3′
	R, 5′-AGCTTCGTATCCCCAATGAGATACA-3′
miR-7-5p	F, 5′-AAAACTGCTGCCAAAACCAC-3′
	R, 5′-GCTGCATTTTACAGCGACCAA-3′
U6	F, 5′-CTCGCTTCGGCAGCACATATACT-3′
	R, 5′-ACGCTTCACGAATTTGCGTGTC-3′
TCF4	F, 5′-CTTCCTCCAAACCAGCAACC-3′
	R, 5′-CCCAACATTCCTGCATAGCC-3′
GAPDH	F, 5′-CGGAGTCAACGGATTTGGTCGTAT-3′
	R:5′-AGCCTTCTCCATGGTGGTGAAGAC-3′
Scramble shRNA sequence	TCCTAAGGTTAAGTCGCCCTC
ANRIL shRNA sequence	GGUCAUCUCAUUGCUCUAU

### Cell transfection

GenePharma Co., Ltd (Shanghai, China) constructed the ANRIL overexpression plasmid (pcDNA-ANRIL), control plasmid (pcDNA-NC), short-hairpin RNA (shRNA) targeting ANRIL (sh-ANRIL), shRNA negative control (sh-NC), miR-7-5p mimics and miR-7-5p inhibitors. Lipofectamine^®^ 2000 (Invitrogen, Carlsbad, CA, USA) was used to perform transfection. ANRIL overexpression plasmid was transfected into MOLT4 cells to establish ANRIL overexpression model. sh-ANRIL was transfected into CCRF-CEM cells and KOPT-K1 cells to construct ANRIL knockdown expression models. The oligonucleotides were transfected into T-ALL cells at a final concentration of 50 nM using the Lipofectamine^®^ 2000 following the manufacturer’s instructions. After transfection for 48 h, the transfection efficiency of cells in each group was detected by qRT-PCR.

### Dual luciferase reporter assay

The target sequence of ANRIL was predicted through bioinformatics analysis. Wild type ANRIL (ANRIL-WT) or mutant ANRIL (ANRIL-MT) sequence was inserted into pmirGLO dual-luciferase miRNA target reporter vector (Promega, Madison, WI, USA). MOLT4 cells, CCRF-CEM cells and KOPT-K1 cells were inoculated in 24-well plates (5000 cells per well), and cultured for 24 h. After that, ANRIL-WT or ANRIL-MT reporter was co-transfected into the cells with miR-7-5p mimics or control microRNA, respectively. Then the cell culture was continued for 48 h, and the luciferase activity of each group was determined using the Dual-Luciferase Reporter Assay System (Promega, Madison, WI, USA).

### Cell proliferation assay

MOLT4 cells, CCRF-CEM cells and KOPT-K1 cells were inoculated in 96-well plates (1000 per well) and cultured. On each day, each well was added with 10 μL Cell Counting Kit-8 (CCK-8; Dojindo, Kumamoto, Japan), and incubated for 2 h. After that, the absorbance of the cells was measured at 450 nm with a microplate reader (Bio-Rad, Hercules, CA, USA). The absorbance of the cells was measured at the 24 h, 48 h, 72 h and 96 h, respectively. Finally, the proliferation curve was plotted.

### Apoptosis assay

FITC Annexin V/Dead Cell Apoptosis Kit (Invitrogen, Shanghai, China) was used to detect the apoptosis of the cells. In brief, MOLT4 cells, CCRF-CEM cells and KOPT-K1 cells were harvested, carefully rinsed twice using PBS and suspended in binding buffer. Next, 5 μL FITC Annexin V kit and 1 μL propidium iodide (PI) solution (100 μg/mL) were used to stain the cells (100 μL of cell suspension) for 30 min at room temperature in the dark. Then the cell apoptosis was detected by flow cytometry (Becton, Dickinson, Mountain View, USA).

### Migration and invasion experiment

The migration experiment was performed with Transwell chambers (8 μm pore diameter, Corning, NY, USA). 24 h after transfection, cells were harvested and resuspended with serum-free medium, and the cell density was modulated to 1 × 10^5^/mL. Then 200 μL of the cell suspension was added into the upper compartment of the chamber, and medium containing 10% FBS was added into the lower compartment. Then the Transwell chamers were moved into the incubator and the cultured was continued. After 24 h, the migrated cells were measured through a microscope. For invasion experiments, Matrigel^®^ (BD, Franklin Lakes, NJ, USA) was coated on the membrane of the Transwell chambers, and the remaining procedures were the same as that in the migration experiment.

### Western blotting

1 × 10^6^ cells were washed with PBS, and lysed using 200 µl RIPA lysis buffer (Beyotime, Hangzhou, China). The supernatant was collected after high speed centrifugation (16,000×*g*, 10 min, 4  °C) and the protein was denatured by heating the samples in boiling water for 5 min. After quantifying the protein by the bicinchoninic acid (BCA) Protein Assay Kit (Boster, Wuhan, China), protein samples were separated by 10% sodium dodecylsulfate polyacrylamide gel electrophoresis (SDS-PAGE) and transferred into a nitrocellulose (NC) membrane (Millipore, MA, USA). Then, the NC membrane was blocked with defatted milk for 30 min at room temperature. Then the primary antibody was added on the NC membrane and incubated overnight at 4 °C. After that, the NC membrane was washed with TBST, followed by being incubated with the secondary antibody for 1 h at room temperature, and then developed chemiluminescence with ECL reagent (Millipore, Bedford, MA, USA). The antibodies used in this study were: anti-TCF4 antibody (ab185736, 1: 500, Abcam, Cambridge, UK), anti-β-actin antibody (ab179467, 1:2000, Abcam, Cambridge, UK), and Goat polyclonal Secondary Antibody to Rabbit IgG–H&L (ab6940, 1:2000, Abcam, Cambridge, UK).

### RNA immunoprecipitation (RIP) assay

RIP was performed using the Magna RIP RNA Binding kit (Millipore, Billerica, MA, USA). Briefly, cells were washed and cross-linked with 0.01% formaldehyde for 15 min. Then the cells were added into the lysates and incubated with the anti-Ago2 antibody conjugated with magnetic beads with rotating overnight at 4  °C. After treating the lysates with proteinase K buffer, immunoprecipitated RNA was extracted by using the RNeasy MinElute Cleanup Kit (Qiagen, Guangzhou, China) and reversely transcribed using Prime-Script RT Master Mix (TaKaRa, Dalian, China). qRT-PCR was used to detect the abundance of miR-7-5p and TCF4.

### Statistical analysis

All data were shown as mean ± SD. SPSS 22.0 software (SPSS Inc., Chicago, IL, USA) and GraphPad Prism 6 software (GraphPad Software Inc., San Diego, CA, USA) was applied to do statistical analysis. Student’s *t*-test method was used to compare the data between two groups. *P *< 0.05 was evaluated as statistically significant.

## Results

### ANRIL and TCF4 were remarkably highly expressed in bone marrow tissues of T-ALL patients, while miR-7-5p was significantly lowly expressed

**Fig. 1:**
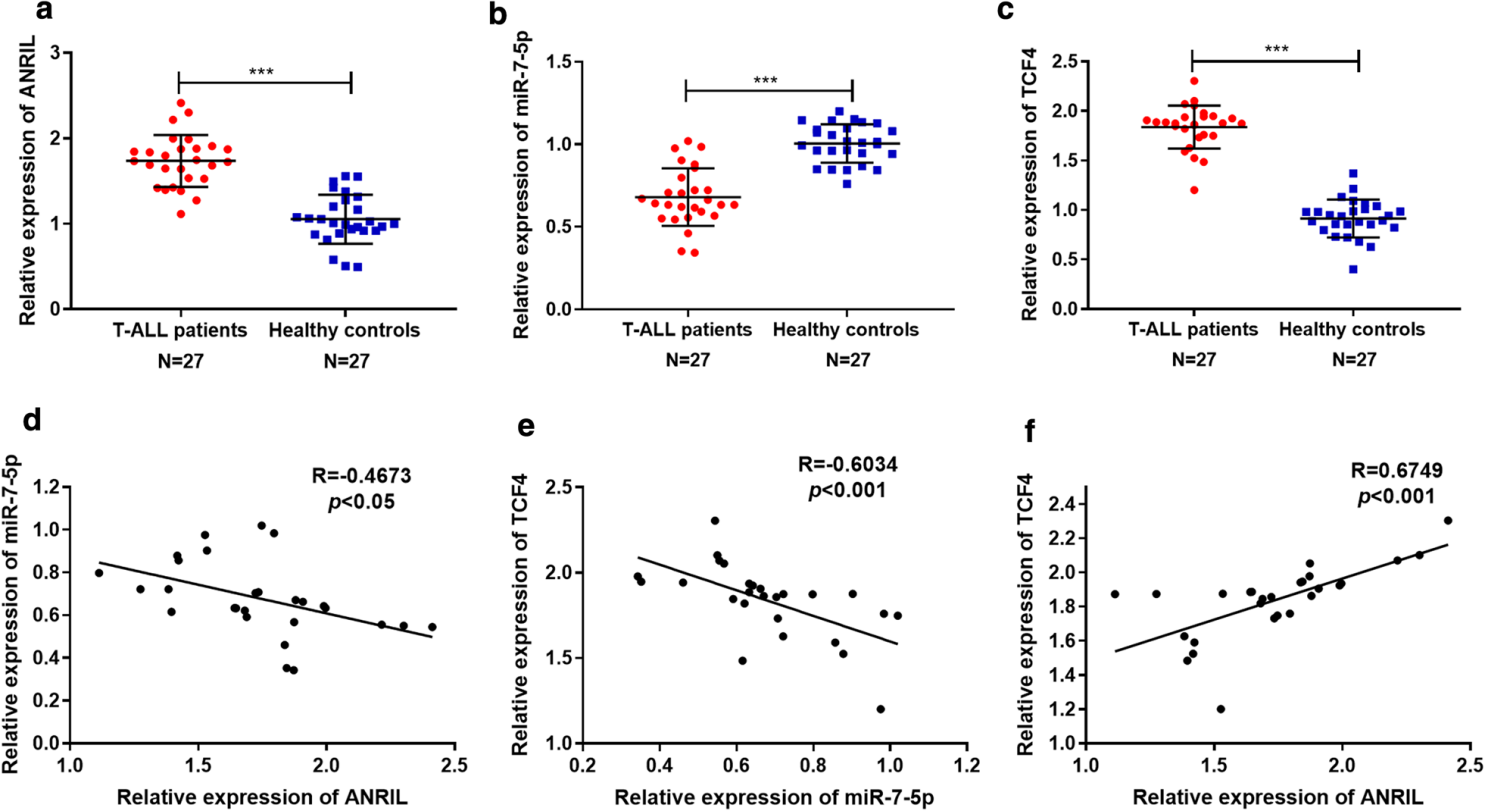
Correlation of ANRIL, miR-7-5p and TCF4 expression levels. **a-c** The relative expression levels of ANRIL, miR-7-5p and TCF4 in bone marrow tissues of 27 T-ALL patients and 27 healthy volunteers were detected by RT-PCR. **d** The expression level of ANRIL in T-ALL was negatively correlated with the expression level of miR-7-5p. **e** The expression level of miR-7-5p was negatively correlated with that of TCF4. **f** The expression level of TCF4 was positively correlated with that of ANRIL. *** *P *< 0.001

To explore the associations among ANRIL, miR-7-5p and TCF4, we firstly used qRT-PCR to detect ANRIL, miR-7-5p and TCF4 mRNA expressions respectively in bone marrow tissues of patients with T-ALL and healthy volunteers. The results suggested that compared with that of healthy controls, the expressions of ANRIL and TCF4 in bone marrow tissue of T-ALL patients were dramatically up-regulated, while miR-7-5p expression was down-regulated (*P *< 0.001, Fig. 1 a–c). Correlation analysis was then performed and revealed a negative correlation between ANRIL and miR-7-5p (R = −0.4673, *P *< 0.05, Fig. 1d); miR-7-5p and TCF4 expressions were also negatively correlated (R = −0.6034, *P *< 0.001, Fig. 1e), while ANRIL expression and TCF4 expression were positively correlated (R = 0.6749, *P *< 0.001, Fig. 1f). These data implied potential regulatory relationships among ANRIL, miR-7-5p and TCF4.

### ANRIL targeted miR-7-5p

**Fig. 2:**
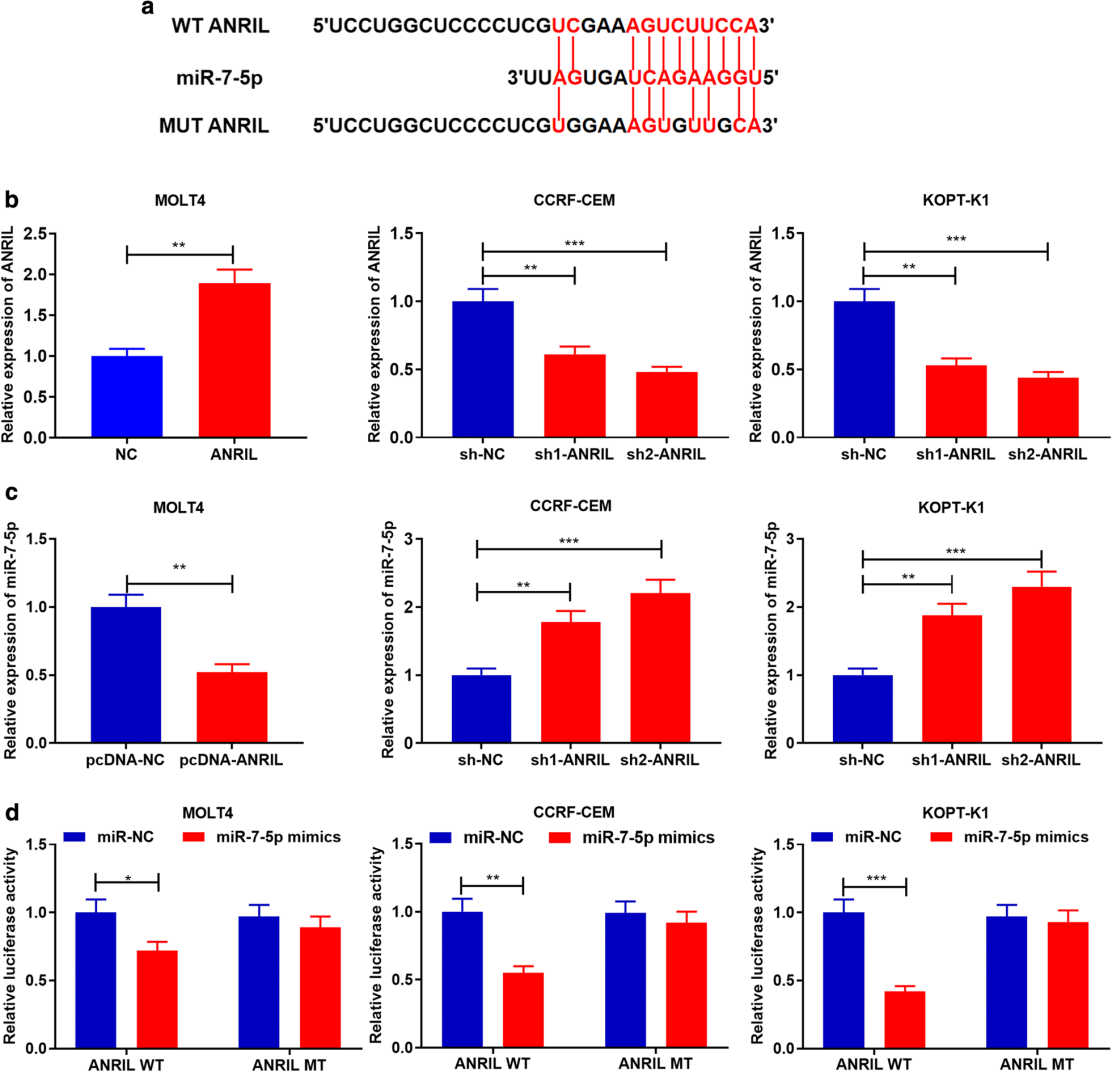
ANRIL targeted miR-7-5p and down-regulated its expression in T-ALL. **a** The binding sequence between ANRIL and miR-7-5p was predicted by bioinformatics analysis. **b** MOLT4 cells were overexpressed with ANRIL, and ANRIL in CCRF-CEM and KOPT-K1 cells was knocked down. **c** ANRIL regulated miR-7-5p expression level in MOLT4 cells, CCRF-CEM cells and KOPT-K1 cells. **d** Dual-luciferase reporter assays were performed in MOLT4, CCRF-CEM and KOPT-K1 cells to validate the binding site between ANRIL and miR-7-5p. *P < 0.05, **P < 0.01, and ***P < 0.001

Then LncBase Predicted v.2 was used to predict the potential target miRNAs of ANRIL. Interestingly, it was found that miR-7-5p was the downstream potential targets for ANRIL, and the potential binding site was presented (Fig. 2a). Compared with T lymphocytes from normal bone marrow tissues, the expression levels of ANRIL in three different T-ALL cells (MOLT4, CCRF-CEM and KOPT-K1) were significantly increased. Of the 3 T-ALL cell lines, ANRIL expression was the lowest in MOLT4 cell lines, which were chosen for the follow-up ANRIL overexpression experiments. ANRIL expression was the highest in CCRF-CEM and KOPT-K1 cell lines, which were selected for succedent ANRIL-knockdown assays (Additional file 1: Figure S1). As shown, ANRIL overexpression plasmid and sh-ANRIL were transfected into T-ALL cells to establish overexpression and knockdown models respectively, and the transfection efficiency was validated by qRT-PCR after transfection for 48 h (*P *< 0.001, Fig. 2b). Next, qRT-PCR showed that overexpression of ANRIL significantly reduced miR-7-5p expression in MOLT4 cells, and ANRIL knockdown enhanced miR-7-5p expression in CCRF-CEM cells and KOPT-K1 cells (*P *< 0.01, Fig. 2c). Additionally, dual luciferase reporter assays confirmed that ANRIL had a binding site for miR-7-5p and probably acted as a “molecular sponge” for miR-7-5p (*P *< 0.05, Fig. 2d).

### ANRIL accelerated proliferation and inhibited apoptosis of T-ALL Cells via regulating miR-7-5p

**Fig. 3:**
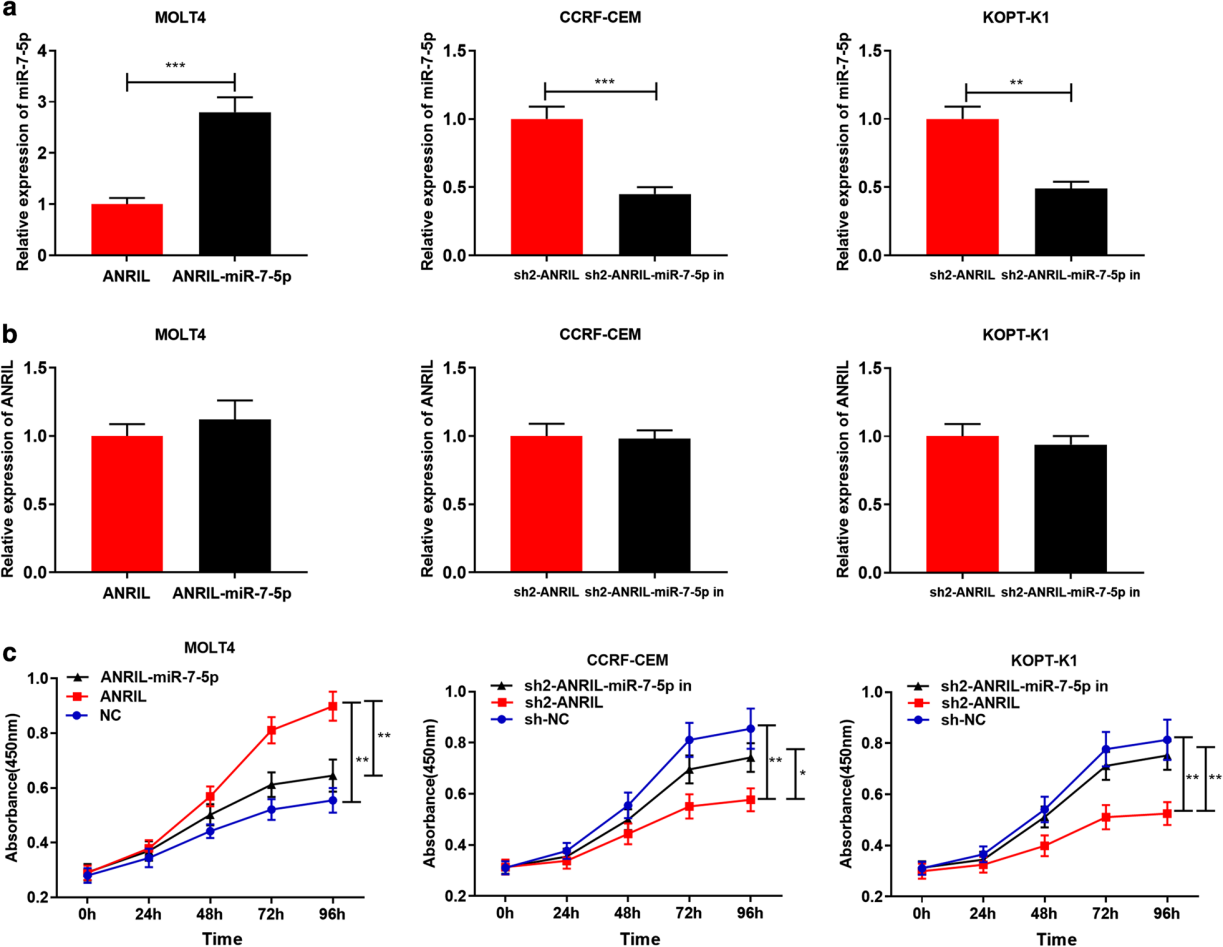
ANRIL promoted proliferation of T-ALL cells by regulating miR-7-5p. **a** Transfection efficiency was detected by RT-PCR after the transfection of miR-7-5p mimics into MOLT4 cells with overexpressed ANRIL, and miR-7-5p inhibitors into CCRF-CEM cells and KOPT-K1 cells with ANRIL knocked down. **b** After transfection, the expression of ANRIL in T-ALL cell lines was detected by RT-PCR. **c** ANRIL promoted MOLT4 cell proliferation and co-transfection of miR-7-5p mimics into MOLT4 cells reversed this effect; ANRIL knockdown inhibited CCRF-CEM and KOPT-K1 cells proliferation, and co-transfection of miR-7-5p inhibitors partly neutralized this effect. *P < 0.05, **P < 0.01, and ***P < 0.001

**Fig. 4:**
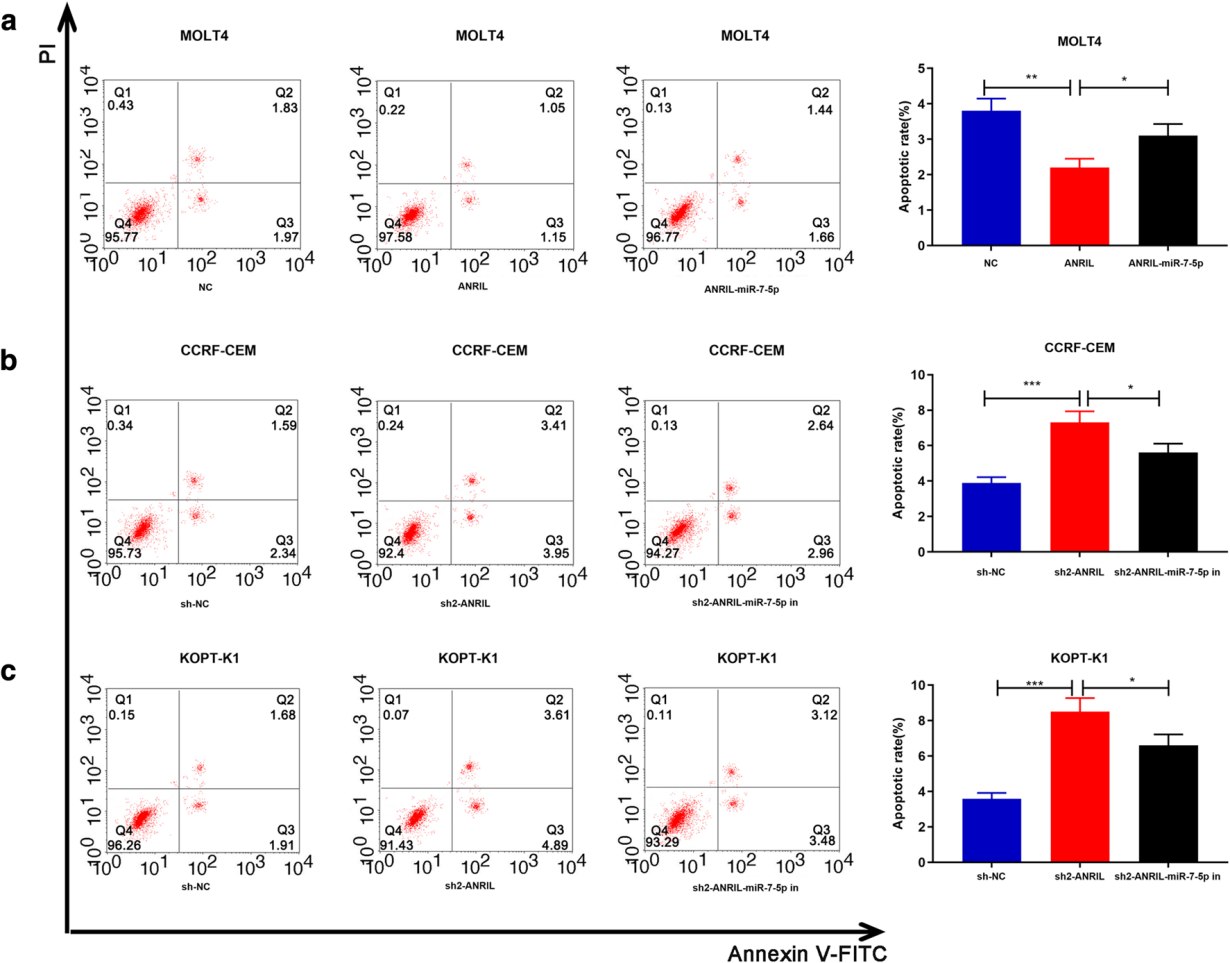
ANRIL Inhibited apoptosis of T-ALL cells by regulating miR-7-5p. **a** ANRIL overexpression inhibited MOLT4 cell apoptosis and co-transfection of miR-7-5p mimics reversed this effect; **b** ANRIL knockdown induced CCRF-CEM cell apoptosis and co-transfection of miR-7-5p inhibitors reversed this effect; **c** ANRIL knockdown induced KOPT-K1 cell apoptosis and co-transfection of miR-7-5p inhibitors reversed this effect. *P < 0.05, **P < 0.01, and ***P < 0.001

To further elucidate the influence of ANRIL and miR-7-5p on proliferation and apoptosis of T-ALL cells, miR-7-5p mimics were co-transfected into MOLT4 cells with ANRIL overexpression; miR-7-5p inhibitors were co-transfected into CCRF-CEM and KOPT-K1 cells with ANRIL knockdown. After transfection for 48 h, the results of qRT-PCR indicated successful transfection (*P *< 0.05, Fig. 3a). It was found that miR-7-5p could not observably change the expression level of ANRIL (*P *> 0.05, Fig. 3b). On this basis, CCK-8 experiment was performed to detect the proliferation of MOLT4, CCRF-CEM and KOPT-K1 cells at 24 h, 48 h, 72 h and 96 h, respectively. It showed that proliferation rate of MOLT4 cells with overexpressed ANRIL was faster than that of that control group (*P *< 0.01); after transfection of the miR-7-5p mimics, this effect was markedly reduced (*P *< 0.01, Fig. 3c). The proliferation rate of CCRF-CEM cells and KOPT-K1 cells with ANRIL knockdown was lower than that of the control group (*P *< 0.01), and this effect was partly reversed by co-transfection of miR-7-5p inhibitors (*P *< 0.05, Fig. 3c). Then we detected the apoptosis of T-ALL cells by flow cytometry after transfection for 48 h. As expected, the apoptotic rate of MOLT4 cells with overexpressed ANRIL was remarkably decreased compared with that of the control group (*P *< 0.01), and this was remarkably reversed by co-transfection of miR-7-5p mimics (*P *< 0.05, Fig. 4a). The apoptosis of CCRF-CEM and KOPT-K1 cells with ANRIL knockdown was significantly higher than that of the control group (*P *< 0.001), and the effects were reduced after transfection of miR-7-5p inhibitors (*P *< 0.05, Fig. 4b–c). These suggested that ANRIL could facilitate the proliferation and inhibit the apoptosis of T-ALL cells partly via regulating miR-7-5p.

### ANRIL promoted migration and invasion of T-ALL cells by regulating miR-7-5p

**Fig. 5:**
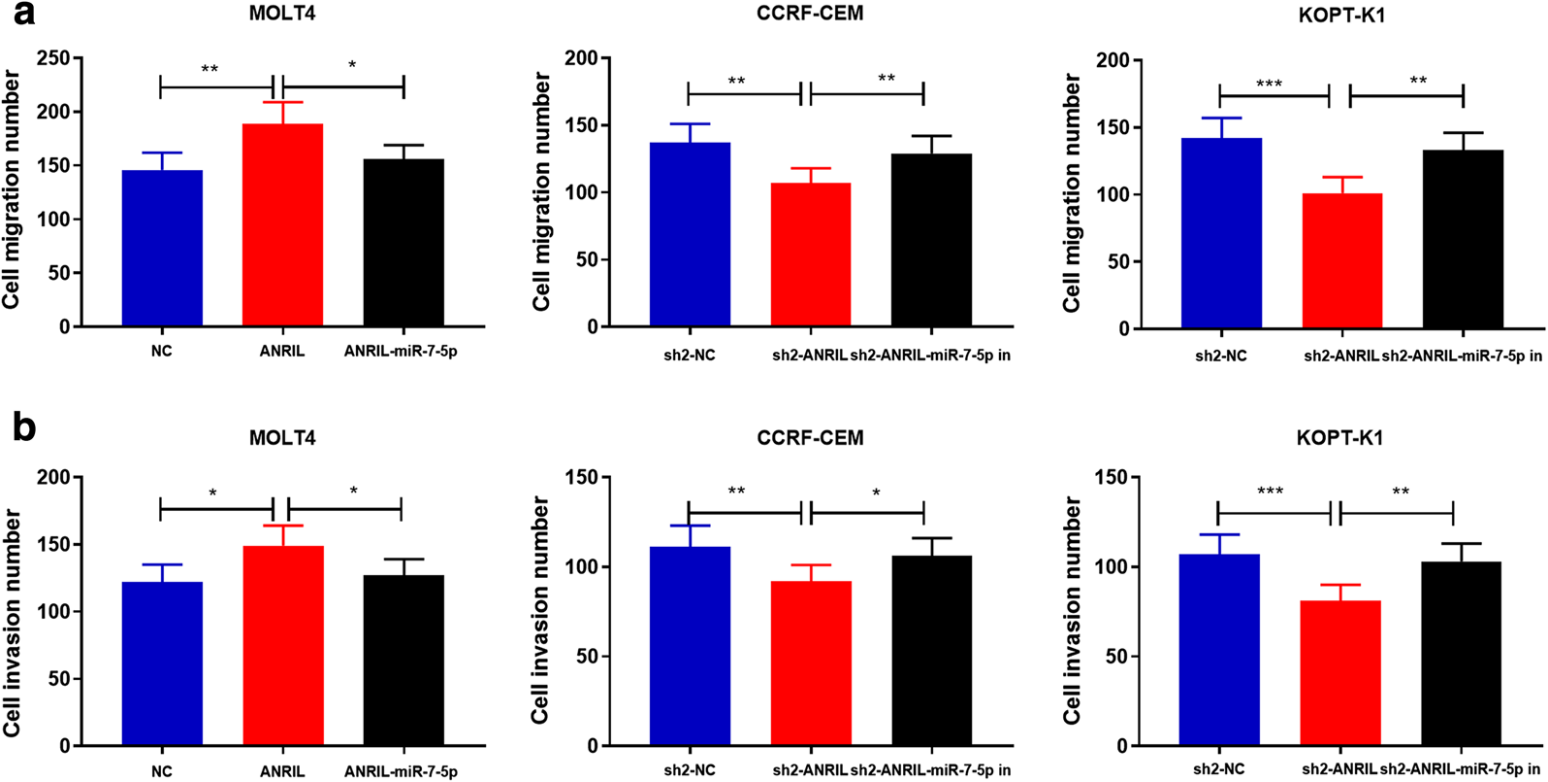
ANRIL promoted T-ALL cells migration and invasion by regulating miR-7-5p. **a** Transwell migration experiment showed that ANRIL overexpression enhanced the migration ability of MOLT4 cells, which could be partially neutralized by miR-7-5p mimics; ANRIL knockdown inhibited the migration of CCRF-CEM and KOPT-K1 cells, and miR-7-5p inhibitors could partially attenuated this. **b** Transwell invasion assay showed that ANRIL overexpression promoted MOLT4 cell invasion, and transfection of miR-7-5p mimics reversed this; ANRIL knockdown inhibited the invasion of CCRF-CEM and KOPT-K1 cells, and miR-7-5p inhibitors partially reversed this. *P < 0.05, **P < 0.01, and ***P < 0.001

Subsequently, we pinpointed the possible mechanisms by which ANRIL regulated the metastasis of T-ALL cells by Transwell assay. As shown (Fig. 5a–b), the number of migration and invasion MOLT4 cells with overexpressed ANRIL was markedly higher than that of the control group (*P *< 0.05); transfection of miR-7-5p mimics partly counteracted the function of ANRIL (*P *< 0.05). Conversely, the ability of migration and invasion of CCRF-CEM cells and KOPT-K1 cells with ANRIL knockdown was impeded (*P *< 0.01), but it could be reversed by co-transfection of miR-7-5p inhibitors (*P *< 0.05). Collectively, these data implied that ANRIL promoted the metastasis of T-ALL cells via regulating miR-7-5p.

### TCF4 was a target of miR-7-5p and could be indirectly modulated by ANRIL

**Fig. 6:**
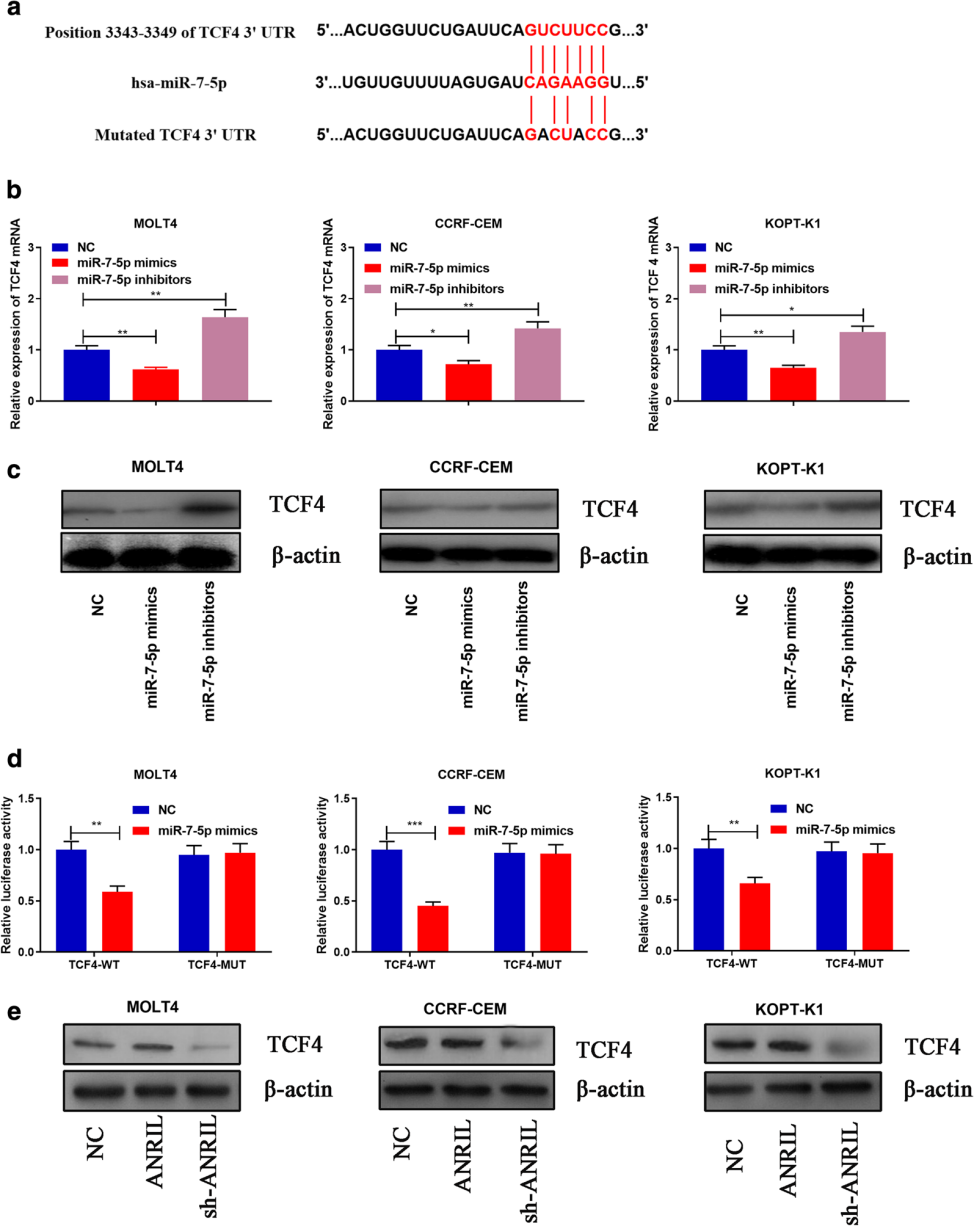
TCF4 was a target gene of miR-7-5p and modulated by ANRIL. **a** Binding site between the 3′UTR of TCF4 and miR-7-5p was predicted by bioinformatics analysis. (**b-c**) RT-PCR and Western blot showed that transfection of miR-7-5p mimics significantly decreased the expression of TCF4 mRNA and protein; conversely, transfection of miR-7-5p inhibitors increased the expression of TCF4 mRNA and protein. **d** MiR-7-5p significantly inhibited the luciferase activity of wild-type TCF4 3′UTR, but had no significant effect on the luciferase activity of the mutated TCF4 3′UTR. **e** Western blot was used to detect the expression of TCF4 after ANRIL was overexpressed or knocked down. *P < 0.05, **P < 0.01, and ***P < 0.001

To fathom the downstream mechanism of ANRIL/miR-7-5p axis, the target genes of miR-7-5p was predicted by multiple online bioinformatics databases (http://www.targetscan.org/vert_72/; http://www.microrna.org/microrna/home.do; http://mirdb.org/miRDB/), and it showed that TCF4 had the potential to be a target of miR-7-5p (Fig. 6a). qRT-PCR and Western blot displayed that TCF4 mRNA and protein expressions were significantly decreased after transfection of miR-7-5p mimics into T-ALL cells, and transfection of miR-7-5p inhibitors had the opposite effect (Fig. 6b–c). Subsequently, dual luciferase reporter experiments revealed that miR-7-5p specifically bound to the 3′UTR of TCF4 (*P *< 0.01, Fig. 6d). Furthermore, findings concluded by RIP suggested the TCF4 mRNA and miR-7-5p were directly interacted (Additional file 2: Figure S2). Western blot also showed that TCF4 expression was obviously up-regulated after transfecting T-ALL cells with ANRIL overexpression plasmid, and the transfection of ANRIL shRNA reduced the expression of TCF4 in T-ALL cells (Fig. 6e). These data implied that TCF4 was a downstream gene of miR-7-5p, and ANRIL could positively regulate its expression indirectly.

## Discussion

ANRIL, located in chromosome 9p21, is confirmed to facilitate cancer progression in a variety of tumors including thyroid cancer, head and neck squamous cell carcinoma, liver cancer, non-small cell lung cancer, cervical cancer and so on [13, 16, 17, 41, 42]. It promotes cancer progression via multiple mechanisms. For example, in thyroid cancer, it activates TGF-β/Smad signaling pathway [13]; in liver cancer, it serves as a competitive endogenous RNA (ceRNA) to promote tumorigenesis through regulation of FGFR1 expression by sponging miR-125a-3p [17]. Its oncogenic role in hematological malignancies has also been revealed in recent years. In acute myeloid leukemia (AML), ANRIL is up-regulated in patients at diagnosis and down-regulated in patients with complete remission; it is proved to modulate the glucose metabolism related pathway of AdipoR1/AMPK/SIRT1 to promote AML cell survival [43]. ANRIL also sponges miR-34a to up-regulate HDAC1, and in turn mediate the epigenetic suppression of ASPP2, which contributes to the proliferation, migration and invasion of AML cells [44]. In this study, it was demonstrated that compared with healthy cases, ANRIL was significantly highly expressed in the bone marrow tissues of T-ALL patients. Our gain-of-function and loss-of-function experiments confirmed that ANRIL overexpression significantly facilitated the proliferation, migration, invasion and inhibited apoptosis of T-ALL cells, while cells with ANRIL knockdown displayed the opposite effect. The above studies indicated that ANRIL was an oncogenic lncRNA during the progression of T-ALL. To our best knowledge, this work was the first to investigate the expression pattern and function of ANRIL in T-ALL.

Recently, the tumor-suppressive effect of the miR-7 family is confirmed in various tumors [45–48]. For example, miR-7 negatively modulates MAP3K9 expression, suppressing the proliferation and promoting apoptosis of pancreatic cancer cells via hindering the MEK/ERK signaling pathway [47]. In addition, miR-7 can regulate TET2, which participates in the pathogenesis of acute myeloid leukemia by disrupting normal hematopoiesis [48]. In this study, miR-7-5p was found to be down-regulated in the bone marrow tissues of T-ALL cells. It was proved that ANRIL could negatively regulate the expression of miR-7-5p in T-ALL cells, and the binding relationship between ANRIL and miR-7-5p was confirmed. It was also demonstrated that the tumor-promoting effects of ANRIL in T-ALL cells were partly reversed by the co-transfection of miR-7-5p. These data indicated that miR-7-5p was a tumor suppressor in T-ALL, and the overexpression of ANRIL contributed to its dysregulation in T-ALL.

As is well-known, TCF4 promotes disease progression in diverse tumors, such as breast cancer, colon cancer and prostate cancer [49–51]. In addition, it is also reported that TCF4 is highly expressed in hematopoietic stem cells, and is associated with the progression of myelodysplastic syndrome and acute myeloid leukemia [52, 53]. In AML, the high expression of TCF4 indicates adverse clinical outcomes of the patients [52]. Additionally, increased TCF4 transcriptional activity contributes to the pathogenesis of transformation of post-myeloproliferative neoplasms into secondary AML, which is related with the abnormal activation of wnt/β-catenin signaling [52–54]. In adult T-cell leukemia, TCF4 up-regulates BIRC5 expression, which probably increase the viability of cell viability [55]. In this work, it was demonstrated that TCF4 was significantly up-regulated in the bone marrow tissues of T-ALL patients. Additionally, it was identified as a target gene of miR-7-5p. It was negatively regulated by miR-7-5p, but positively regulated by ANRIL. These data suggested that ANRIL/miR-7-5p/TCF4 axis was involved in the tumorigenesis and progression of T-ALL.

This work has several limitations. First of all, in vivo experiments are of great significance to further validate the role of ANRIL/miR-7-5p/TCF4 axis in T-ALL progression. Additionally, whether ANRIL can regulate other phenotypes of T-ALL cells (such as chemosensitivity) awaits further exploration. Last but not the least, a considerable number of patients from different centers is required, and it is worth exploring whether the dysregulation of ANRIL/miR-7-5p/TCF4 axis is associated with the patients’ prognosis.

## Conclusion

To sum up, this study confirms that ANRIL promotes the proliferation and metastasis of T-ALL cells via modulating miR-7-5p and TCF4. This work provides a new theoretical basis for clarifying the mechanism of NPC progression, and implies that ANRIL, miR-7-5p and TCF4 are potential diagnostic biomarkers and therapy targets.

## Supplementary information

**Additional file 1: Figure S1.** RT-PCR was used to measure the expression of ANRIL in thymocyte, MOLT4, CCRF-CEM and KOPT-K1 cell lines.

**Additional file 2: Figure S2.** RIP assay confirmed the binding between TCF4 and miR-7-5p in MOLT4, CCRF-CEM and KOPT-K1 cell lines.

## Data Availability

The data bracing the results of this survey can be acquired from the corresponding author as required.

## Abbreviations

T-ALL: T-cell acute lymphoblastic leukemia
ANRIL: Antisense non-coding RNA in the INK4 locus
RT-PCR: Real-time polymerase chain reaction
CCK-8: Cell counting kit-8
TCF4: Transcription factor 4
lncRNAs: Long non-coding RNAs
miRNAs: microRNAs
3′-UTR: 3′-untranslated region
FBS: Fetal bovine serum
ANRIL-WT: Wild type ANRIL
ANRIL-Mut: Mutant type ANRIL
