# Supplementary figures and images for: LncRNA ANRIL/miR-7-5p/TCF4 axis contributes to the progression of T cell acute lymphoblastic leukemia
Source: Cancer Cell Int. 2020 Jul 23;20:335. doi: 10.1186/s12935-020-01376-8 (PMC7376839; doi:10.1186/s12935-020-01376-8)

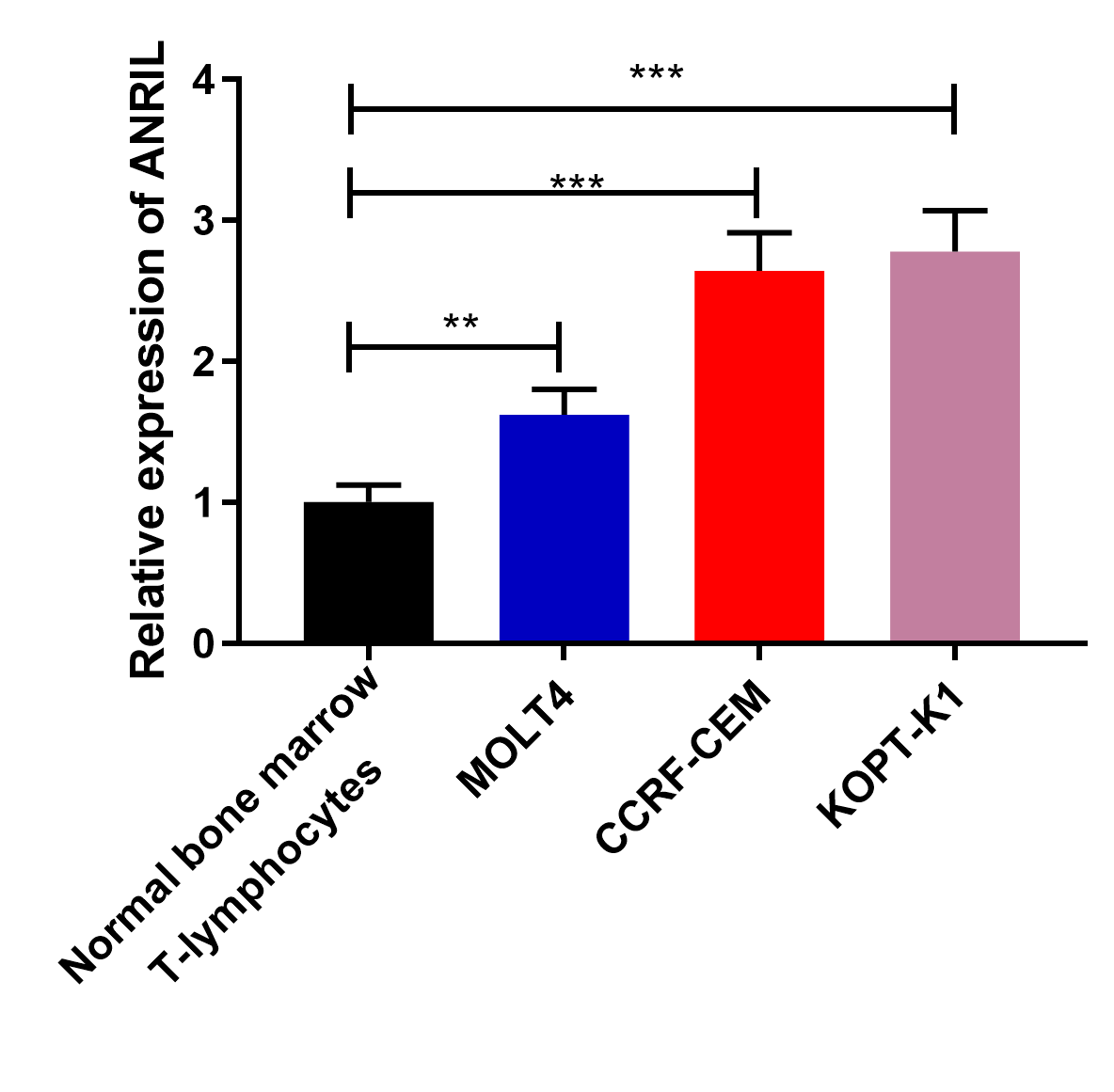

Supplement: Supplementary file 1 — Additional file 1: Figure S1. RT-PCR was used to measure the expression of ANRIL in thymocyte, MOLT4, CCRF-CEM and KOPT-K1 cell lines. [file 12935_2020_1376_MOESM1_ESM.tif]

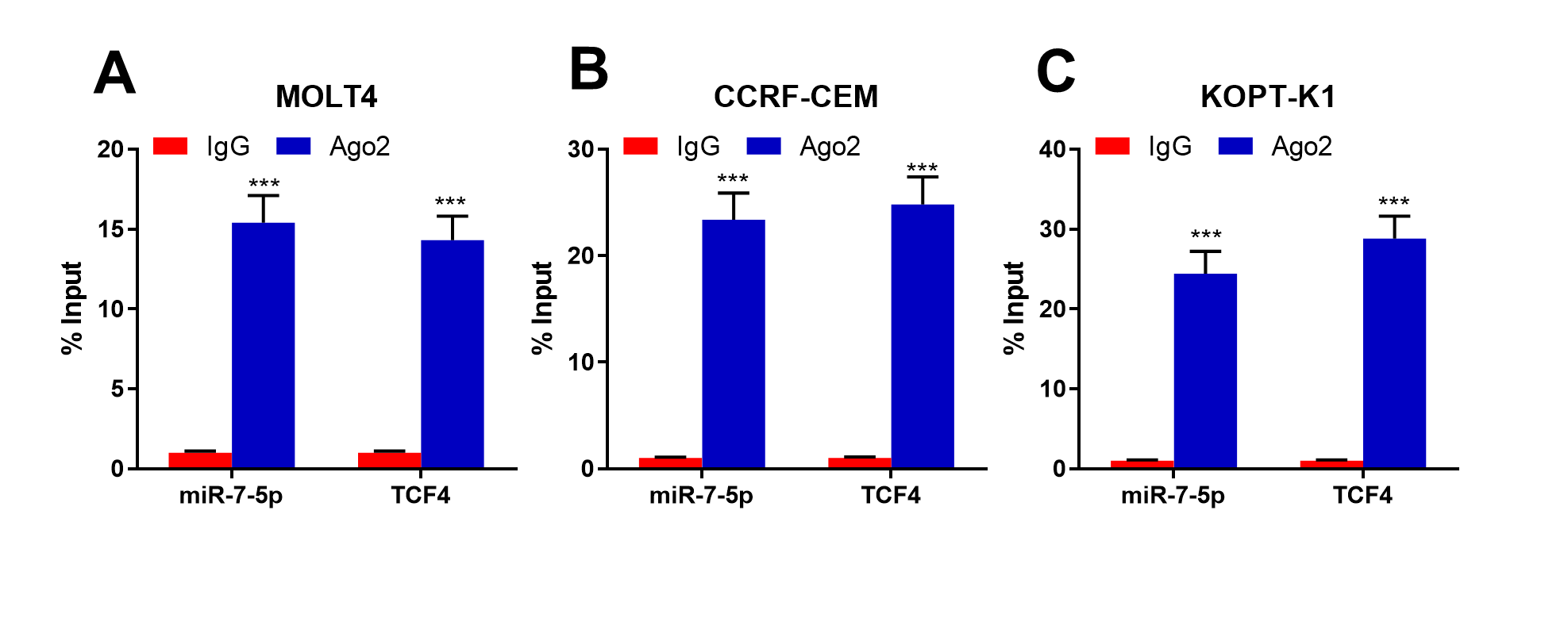

Supplement: Supplementary file 2 — Additional file 2: Figure S2. RIP assay confirmed the binding between TCF4 and miR-7-5p in MOLT4, CCRF-CEM and KOPT-K1 cell lines. [file 12935_2020_1376_MOESM2_ESM.tif]
